# Retiring Allowances to Charity Officials

**Published:** 1890-05-10

**Authors:** 


### Passing Topics.

### RETIRING ALLOWANCES TO CHARITY OFFICIALS.

The appearance of a recent article under this heading has brought a timely reminder that the praiseworthy designs of the promoters of the National Pension Fund for Nurses are more far-reaching than appears upon the surface, and that the constitution of the society provides for the inclusion of all officials, male or female, employed in any medical institution.

That being so, perhaps we may be permitted to hope for a revision of the title. Sentiment governs conduct more or less in all walks of life, and while there are, doubtless, many officials not of the class named who would be glad to share in the advantages of the society, few would view with indifference the fact that their prospect of a pension was to be derived from a society ostensibly established for the benefit of the nurses. Still, notwithstanding this drawback, we learn that although no publicity has been given as yet to this portion of the contemplated work, several of the higher officials of charities, including secretaries, medical officers, and lady superintendents, have already joined. This is conclusive testimony, but even had it not been forthcoming the value of the proposal could scarcely have been questioned, while its practicability is exhibited in the fact that a similar system obtains in connection with many banks and other large commercial establishments.

We are aware that it has pleased certain critics to object that the premiums are upon too high a scale, and to intimate that the nurses, for all the advantage they will get out of the Pension Fund, might just as well go at once to an insurance office. This is a complaint of which the truth is easily tested. Obviously there can be no wide difference between the premiums chargeable for the same services by any two societies competing for clients among the same classes, and equally careful of their credit and solvency. The premiums payable are deduced from calculations all but mathematically correct, and their substantial accuracy is demonstrated by the fact that though the methods of different societies may not be precisely alike, the result arrived at is practically the same in all cases. It is as difficult, therefore, to comprehend the competency of the criticism which disparages as excessive a payment experience has shown to be no more than necessary, as to understand its bona fides when the mention of certain material factors, which cannot be overlooked in determining the value of the boon, is altogether suppressed.

Foremost amongst these are, first, the Bonus Fund, by means of which the pensions of nurses are to be increased beyond the amount actually paid for; and secondly, the recommendation, amounting almost to a stipulation, that the hospitals should contribute one-half of the premiums by way of contracting themselves out of the obligation to pay pensions to retired nurses, or, to put it more pleasantly, in order to show their appreciation of services rendered, and to encourage thrift.

Neither of these is a mere suggestion potential only in possibilities. The Bonus Fund, thanks in chief measure to the benefactions of four generous men and to the industry and consummate skill with which the idea has been fostered, already amounts to the substantial total of £40,000, while several hospitals, among them the London and Guy's, have affiliated themselves to the fund, in the interests of the nurses.

As regards the Bonus Fund, its operations are confined to the nurses, and no one will begrudge the latter that advantage over other officials, but it is proposed that the payment of one half of the premiums shall apply to all alike. Of course to make provision for the higher grade officers is a much more serious matter individually than to do the same for the nurses. Even in regard to the nurses, the question arises, what is a fair and sufficient pension? Fifteen pounds a year, the amount apparently most favoured, does not appear a very liberal provision for old age, but the action of the Bonus Fund will, in their case, materially increase the annuity receivable. As regards the others, the fact that the individual pension must be a heavier charge upon the institution is an additional reason why advantage should be taken of the opportunity offered by the society. An equitable arrangement made at the outset of an officer's career, between himself and the hospital, whereby he is relieved from all anxiety about that period of enforced inactivity which comes sooner or later to all of us, cannot but be advantageous to both parties, while if it should happen that payments are discontinued, the sum already contributed in premiums is returnable to him with moderate interest, or if he die, a like return would be made to his family.

Our contention was and is that an officer of any grade who has worked a sufficient number of years, suppose we say not less than twenty, for the same institution may fairly ask of it a retiring allowance of some sort when age or infirmity no longer allows him to continue his office, and we welcome the overtures of this society because they seem calculated to facilitate a more general performance of an obvious but not always adequately acknowledged duty. Agricola.

